# White Matter Integrity Underlies the Physical-Cognitive Correlations in Subjective Cognitive Decline

**DOI:** 10.3389/fnagi.2021.700764

**Published:** 2021-08-02

**Authors:** Yi-Chia Wei, Chih-Chin Heather Hsu, Wen-Yi Huang, Yao-Liang Chen, Chemin Lin, Chih-Ken Chen, Chen Lin, Yu-Chiau Shyu, Ching-Po Lin

**Affiliations:** ^1^Institute of Neuroscience, National Yang Ming Chiao Tung University, Taipei, Taiwan; ^2^Community Medicine Research Center, Chang Gung Memorial Hospital, Keelung, Taiwan; ^3^Department of Neurology, Chang Gung Memorial Hospital, Keelung, Taiwan; ^4^Center of Geriatrics and Gerontology, Taipei Veterans General Hospital, Taipei, Taiwan; ^5^College of Medicine, Chang Gung University, Taoyuan, Taiwan; ^6^Department of Radiology, Chang Gung Memorial Hospital, Keelung, Taiwan; ^7^Department of Medical Imaging and Radiological Sciences, Chang Gung University, Taoyuan, Taiwan; ^8^Department of Psychiatry, Chang Gung Memorial Hospital, Keelung, Taiwan; ^9^Department of Biomedical Sciences and Engineering, National Central University, Taoyuan, Taiwan; ^10^Department of Nursing, Chang Gung University of Science and Technology, Taoyuan, Taiwan; ^11^Institute of Molecular Biology, Academia Sinica, Taipei, Taiwan; ^12^Department of Biomedical Imaging and Radiological Sciences, National Yang Ming Chiao Tung University, Taipei, Taiwan; ^13^Aging and Health Research Center, National Yang Ming Chiao Tung University, Taipei, Taiwan; ^14^Brain Research Center, National Yang Ming Chiao Tung University, Taipei, Taiwan

**Keywords:** subjective cognitive decline, cognitive performance, physical activity, actigraphy, mediation effects, diffusion tension imaging, white matter, community-dwelling older adults

## Abstract

**Objective:** Although previous studies postulated that physical and cognitive decline codeveloped in preclinical dementia, the interconnected relationship among subjective cognitive complaints (SCCs), objective cognitive performance, and physical activity remained hazy. We investigated the mediating roles of physical activity between subjective and objective cognition. Diffusion tensor imaging (DTI) was utilized to test our hypothesis that brain white matter microstructural changes underlie the physical-cognitive decline in subjective cognitive decline (SCD).

**Methods:** We enrolled cognitively normal older adults aged > 50 years in the Community Medicine Research Center of Keelung Chang Gung Memorial Hospital during 2017–2020. Regression models analyzed mediation effects of physical activity between subjective and objective cognition. The self-reported AD8 questionnaire assessed SCCs. The SCD group, defined by AD8 score ≥ 2, further underwent diffusion MRI scans. Those who agreed to record actigraphy also wore the SOMNOwatch™ for 72 h. Spearman's correlation coefficients evaluated the associations of diffusion indices with physical activity and cognitive performance.

**Results:** In 95 cognitively normal older adults, the AD8 score and the Montreal Cognitive Assessment (MoCA) score were mediated partially by the metabolic equivalent of the International Physical Activity Questionnaire-Short Form (IPAQ-SF MET) and fully by the sarcopenia score SARC-F. That is, the relation between SCCs and poorer cognitive performance was mediated by physical inactivity. The DTI analysis of 31 SCD participants found that the MoCA score correlated with mean diffusivity at bilateral inferior cerebellar peduncles and the pyramids segment of right corticospinal tract [*p* < 0.05, false discovery rate (FDR) corrected]. The IPAQ-SF MET was associated with fractional anisotropy (FA) at the right posterior corona radiata (PCR) (*p* < 0.05, FDR corrected). In 15 SCD participants who completed actigraphy recording, the patterns of physical activity in terms of intradaily variability and interdaily stability highly correlated with FA of bilateral PCR and left superior corona radiata (*p* < 0.05, FDR corrected).

**Conclusions:** This study addressed the role of physical activity in preclinical dementia. Physical inactivity mediated the relation between higher SCCs and poorer cognitive performance. The degeneration of specific white matter tracts underlay the co-development process of physical-cognitive decline in SCD.

## Introduction

According to the National Institute on Aging and the Alzheimer's Association guideline, subjective awareness of subtle cognitive decline occurs at the preclinical stage of Alzheimer's disease (Sperling et al., 2011). Subjective cognitive decline (SCD) is defined by presence of subjective cognitive complaints (SCCs) and absence of cognitive impairment or neuropsychiatric deficits. In SCD, compensatory mechanisms preserve the cognitive performance, and the severe cognitive decline has not yet begun (Jessen et al., 2014).

Previous studies separately indicated that SCCs and weakened physical conditions are the early warning signs of cognitive deterioration in preclinical dementia. SCCs identified the risk of cognitive decline in older adults, that is, people with memory complaints had a 6.67% annual conversion rate to mild cognitive impairment (MCI) and a relative risk of dementia of 2.07, compared with those without SCCs (Mitchell et al., 2014). On the other hand, slowing down of gait speed was observed a decade earlier than the occurrence of MCI (Buracchio et al., 2010). A community study of non-demented older adults also found the relationship between impaired cognitive performance and physical frailty (Wu et al., 2015). Physical frailty has been reported positively associated with the decline of both subjective and objective cognition in older non-demented women (Gifford et al., 2019). Although an interconnected triangle relationship seemed to emerge among physical activity, subjective cognition, and objective cognition, the causal relationship among these three aspects remained hazy.

Several longitudinal studies on preclinical dementia and normal older adults attempted to clarify the roles of either SCCs or weakened physical conditions in developing cognitive impairment. SCCs were deemed as risk factors in subsequent cognitive impairment (Hohman et al., 2011; Amariglio et al., 2015a; Kaup et al., 2015). On the other hand, physical activity (Buchman et al., 2012; Tan et al., 2017) and physical frailty (Auyeung et al., 2011) predict future cognitive decline in cognitively normal older adults. Both SCCs and a slow gait co-play a predictive role in the development of dementia and have been described as “motoric cognitive risk syndrome” (Verghese et al., 2013). From the above review, the physical-cognitive and subjective-objective cognition relationships are straightforward. However, the interaction between SCCs and physical inactivity along the cognitive decline trajectories remains unclear. Whether physical inactivity has additive effects on the pathway of subjective-objective cognitive decline is unknown. Therefore, we hypothesized that physical activity mediated between the presence of SCCs and worsening of cognitive performance, and tested the hypothesis in community-dwelling older adults.

Diffusion MRI is a sensitive and quantitative tool to detect white matter microstructure change (Alexander et al., 2011). Previous studies found the relationship of either cognitive functions (Roberts et al., 2013) or physical activity (Strommer et al., 2020) to white matter microstructure. The pathology of Alzheimer's disease includes myelin loss and oligodendrocyte damage (Nasrabady et al., 2018). Diffusion MRI has shown its ability to reveal the hierarchically white matter changes in the disease spectrum from SCD, MCI, to Alzheimer's disease (Doan et al., 2017). The white matter microstructure changes were both globally (Ohlhauser et al., 2019) and locally distributed in SCD, especially at the parahippocampal white matter (Wang et al., 2012). On the other hand, diffusion MRI studies also revealed the associations between physical activity and white matter microstructure. In healthy young adults, high fractional anisotropy (FA) in widespread white matter mediated the physical fitness and enhanced cognitive performance (Opel et al., 2019). Anomalies in white matter and decreased daily activity may correlate with lower motor function in healthy older adults (Fleischman et al., 2015). Furthermore, greater white matter volume, less white matter lesions, and relatively intact white matter microstructure are associated with a higher physical activity level (Sexton et al., 2016). Therefore, we used diffusion MRI to discover the white matter changes underlying the physical-cognitive correlations in SCD.

This study aimed to extend our knowledge of the interconnected relationship among physical activity, subjective cognition, cognitive performance, and underlying white matter microstructure change in cognitively normal older adults. To this end, we recruited cognitively normal older adults from the communities and underwent diffusion tensor imaging (DTI) study in those participants with SCD. First, we hypothesized that physical inactivity mediates the relation of increased SCCs to decreased cognitive abilities. We tested the hypothesis, using a regression-based mediation analysis in all enrolled participants. Second, we assumed that the white matter abnormalities interrupt the neuronal connections and manifest as impaired physical and cognitive conditions; that is, white matter disintegrity underlies the physical-cognitive decline could be observed by the correlations of DTI indices to physical activity and cognition performance in the participants with SCD.

## Materials and Methods

### Participant Enrollment

The Community Medicine Research Center of Keelung Chang Gung Memorial Hospital recruited residents aged > 50 years from December 2017 to August 2020. The healthy participants were enrolled with the following criteria: free of major organ failure, dementia, other neurological diseases, and brain surgeries. The Mini-International Neuropsychiatric Interview excluded the participants with psychiatric disorders (Sheehan et al., 1998). All the participants underwent structural MRI scans of the brain, and those with brain lesions were excluded from the enrollment.

Definition of SCD followed the criteria of the Subjective Cognitive Decline Initiative Working Group, which entails: (1) a self-experienced persistent decline in cognitive capacity in comparison with a previously normal status and not related to an acute event, and (2) expected performance on standardized cognitive tests (Molinuevo et al., 2017). The diagnosis of SCD was based on both cognitively normal and having SCCs by ≥2 points of a self-reported AD8 score (Galvin et al., 2006; Yang et al., 2011; Wei et al., 2019). The definition of cognitive impairment was the Montreal Cognitive Assessment (MoCA) score lower than one standard deviation from the age and education-matched normative standards (Rossetti et al., 2011). The diagnostic criteria of MCI were independent daily activity, non-demented, but having cognitive impairment (Petersen et al., 1999). After excluding those who fulfilled the criteria of MCI, we divided the participants into SCD and normal control (NC) groups. The SCD group further underwent diffusion MRI scans. Those SCD participants who agreed to have physical activity monitoring recorded 72 h of actigraphy by the SOMNOwatch™ (SOMNOmedics, Randersacker, Germany).

This study was approved by the Institutional Review Board of Chang Gung Memorial Hospital (No. 201600580B0, 201600270B0, and 200600269B0). All the participants provided written informed consent before entering the study. The identifier of the cohort on the *ClinicalTrials.gov* was NCT04839796, entitled the “Northeastern Taiwan Community Medicine Research Cohort” (NTCMRC).

### Measurements

The AD8 questionnaire recorded SCCs by posing eight questions to measure the personal experience of changes in daily cognitive function, including impairment of judgment, loss of interest in hobbies or activities, repetition of the same things (stories, questions, or statements), trouble learning with using tools (appliances or gadgets), forgetting correct month or year, difficulties in handling complicated financial affairs, problems remembering appointments, and consistent issues with thinking and memory. Confirmation of each cognitive change added 1 point to the total score. An AD8 score ≥ 2 points was considered significant decline in subjective cognition (Galvin et al., 2005). The traditional Chinese version of AD8 verified in Taiwan (Yang et al., 2011) was used as a self-reported assessment in this study (Wei et al., 2019).

The structured cognitive test MoCA evaluated orientation, short-term memory, numerical calculations, visuospatial skills, and executive functions of participants. The traditional Chinese version of MoCA used in this study had been validated in Taiwan (Tsai et al., 2012). Other cognitive tests in this study included the Mini-Mental State Examination (MMSE), digit symbol-coding (DSC), digit span test (DST), letter-number sequencing (LNS), category fluency (CF), and facial memory test (FMT).

SARC-F was a simple questionnaire used for rapid diagnosis of sarcopenia. SARC-F scores ranged from 0–10 by 0–2 increments for each of the five components: strength, assistance in walking, rising from a chair, climbing stairs, and falls. Score ≥ 4 predicted sarcopenia (Malmstrom et al., 2016).

The International Physical Activity Questionnaire-Short Form (IPAQ-SF) was a subjective measure of physical activity based on four generic items: vigorous-intensity physical activity, moderate-intensity activity, walking, and sitting. IPAQ-SF scoring could be transformed into metabolic equivalent of task (MET) minutes per week or categorized by low, moderate, or high levels (Hagstromer et al., 2006).

Actigraphy was an objective detection of physical activity. The SOMNOwatch™ quantitatively recorded activity intensity, duration, and frequency and analyzed movement by a body position sensor and three accelerometers of x, y, z-axis. A sensor of ambient light on the device and the manual markers of the users determined the differentiation of sleep/wake activity. The SOMNOwatch™ had been validated in studying sleep medicine (Dick et al., 2010) and movement disorders (Bove et al., 2018) and served as an objective measure of physical activity (Lang et al., 2013). In this study, the participants wore the SOMNOwatch™ over their non-dominant hands for 72 h. The raw data were transformed into measurable variables to quantify circadian rhythm: intradaily variability and interdaily stability (Sokolove and Bushell, 1978; Van Someren et al., 1999; Cespedes Feliciano et al., 2017). Low intradaily variability or high interdaily stability represented a better pattern of physical activity.

### Mediation Analysis of Physical Conditions Between SCCs and Cognitive Performance

The regression analysis tested the mediation effects of physical conditions between SCCs and cognitive performance. The AD8 score was the independent variable (X), while the MoCA score was the dependent variable (Y). Mediators (M) to be tested were the SARC-F score and the IPAQ-SF MET. Structural equations measured the total, direct, and indirect effects of X on Y to analyze the mediation effects of M. The mediation model (model 4) in the PROCESS macro (Hayes, 2018) was performed on the SPSS version 20 (International Business Machines Corporation, New York, United States), with a level of confidence at 95 and a number of bootstrap samples of 5,000.

### Diffusion Image Preprocessing

All MRI images were acquired on the SIEMENS MAGNETON Skyra 3T scanner (Siemens Healthineers, Germany) in the Chang Gung Memorial Hospital, Keelung, Taiwan. All the participants underwent the MRI protocol of T1-weighted (T1W) image. The SCD group participants who agreed to undergo diffusion MRI study further underwent scanning of a set of DTI. T1W image was scanned, using magnetization-prepared rapid acquisition with gradient echo (MPRAGE) protocol with TR/TE/TI = 2,200/2.45/900 ms, a flip angle = 8°, voxel size = 1 × 1 × 1 mm^3^ without an interslice gap, FOV = 256 × 256 × 176. DTI data were acquired, using single-shot spin-echo echo-planar images with TR/TE = 7,600/83 ms, voxel size = 2 × 2 × 2 mm^3^, matrix size = 128 × 128 × 62, 64 non-collinear gradient directions with a b-value of 1,000 s/mm^2^, three null images (b = 0 s/mm^2^), one excitation, and one inverse phase-encoding image for the correction of susceptibility-induceddistortions.

MRI data were all processed, using FSL v5.0.9 (Functional MRI of the Brain Software Library, http://fsl.fmrib.ox.ac.uk). DTI data first underwent corrections of susceptibility-induced distortion, head motion, and eddy current distortion, using topup and eddy commands. The diffusion tensor model was fitted in each voxel, and the quantitative diffusion maps, including FA and mean diffusivity (MD), were calculated *via* DTIFIT command. To extract the regional white matter microstructure status, we leveraged the tract-based spatial statistics (TBSS) pipeline implemented in FSL. The sequential steps were as follows: (1) non-linear registration of FA maps onto the 1 × 1 × 1 mm FMRIB58_FA template, (2) creation of a cross-participant mean FA skeleton from all the participants with a threshold of FA = 0.2, (3) applying the non-linear warp on the other diffusion maps, (4) calculating the averaged diffusion value of each white matter area based on 48 white matter labels defined by the Johns Hopkins University's atlas (JHU ICBM DTI-81 atlas) (Mori et al., 2008, 2009; Oishi et al., 2008), and (5) applying the resulting diffusion values of each white matter area to the subsequent statistical analysis.

### Correlation Analysis

Spearman's correlation coefficient (*r*_*s*_) measured the association strength of the diffusion indices to the subjective and objective physical activity indices and cognitive scores, respectively. The FA and MD values of the 48 white matter tracts of the JHU ICBM DTI-81 atlas were tested for correlations with the AD8 score, MoCA score, IPAQ-SF MET, SARC-F score, actigraphy intradaily variability, and interdaily stability. An uncorrected *p* < 0.05 was considered statistically significant. All the *p-*values further underwent type I error corrections for multiple comparisons by false discovery rate (FDR) correction.

Partial correlation analysis was further utilized to eliminate the confounding effect between physical activity and cognitive performance. The cognition-related correlations were corrected by the corresponding diffusion index of the physical activity-related tracts; vice versa, we controlled the cognition-related tracts to test the physical activity-related correlations. A partial correlation coefficient (*r*_*p*_) was considered statistically significant at *p* < 0.05.

## Results

### Initial Participant Enrollment

**Figure 1:**
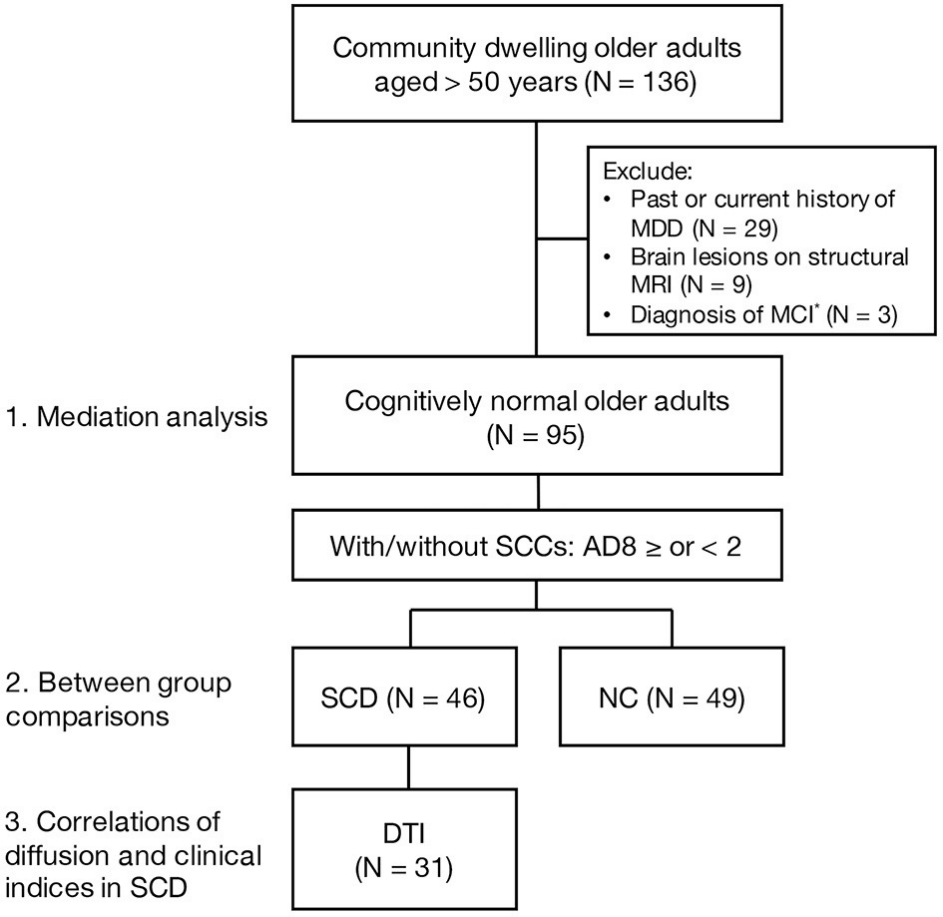
A flow chart of enrollment. MDD, major depressive disorder; MCI, mild cognitive impairment; SCCs, subjective cognitive complaints; SCD, subjective cognitive decline; NC, normal control; DTI, diffusion tension imaging. *MCI diagnosis was based on the MoCA score lower than one standard deviation below the age- and sex-adjusted norms.

A total of 136 community-dwelling older adults were screened for enrollment. Through the Mini-International Neuropsychiatric Interview, 29 participants with a past or current history of major depressive disorder were excluded. Nine participants were excluded for brain lesion(s) on structural MRI, and so were the other three participants diagnosed with MCI. A final number of 95 participants were enrolled with a mean age of 65.64 ± 5.56 (a range, 52–83; median, 66) years, a female-to-male ratio of 1.42, and education years of 9.71 ± 4.21 (a range, 0–18; median, 9) years (Figure 1).

### Mediation Effects of Physical Activity Between Subjective and Objective Cognition in Cognitively Normal Older Adults

**Figure 2:**
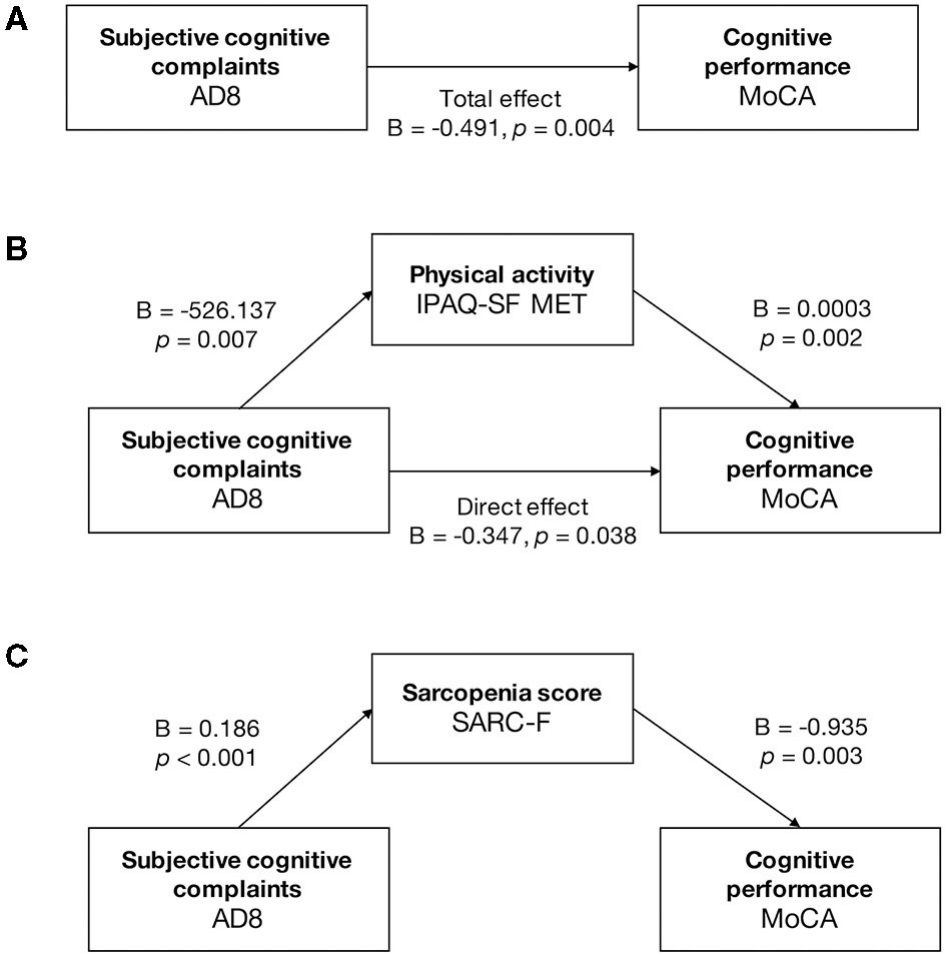
Mediation analysis in cognitively normal older adults. Mediation analysis was run on the PROCESS model 4, with a level of confidence for all confidence intervals of 95.0000 and 5,000 bootstrap samples for percentile bootstrap confidence intervals. **(A)** The total effect between the AD8 and MoCA score was negative and significant (B = −0.491, *p* = 0.004). **(B)** The IPAQ-SF MET partially mediated between the AD8 and the MoCA. The AD8 score caused the MoCA score directly (direct effect: B = −0.347, 95% CI −0.673 to −0.021, *p* = 0.038) and partially through the IPAQ-SF MET (indirect effect: B = −0.144, 95% CI −0.291 to −0.043). **(C)** The SARC-F had full mediation effects between the scores of AD8 and MoCA, with significant indirect effect (B = −0.173, 95% CI −0.411 to −0.037) and nonsignificant direct effect (B = −0.317, 95% CI −0.652 to 0.017, *p* = 0.062).

The structured regression model revealed the mediation effects of physical activity between subjective cognition and objective cognitive performance (Figure 2). The AD8 score had a negative and significant total effect on the MoCA score [B = −0.491, standard error (SE) = 0.165, *p* = 0.004] (Figure 2A). The IPAQ-SF MET was a partial mediator between the AD8 and the MoCA score by significant indirect effect [B = – 0.144, SE = 0.063, 95% confidence interval (CI) −0.291 to −0.043] and direct effect (B = −0.347, SE = 0.164, 95% CI −0.673 to −0.021) (Figure 2B). The AD8 score caused the MoCA score completely through the SARC-F score (indirect effect B = −0.173, SE = 0.097, 95% CI −0.411 to −0.037) (Figure 2C).

### Physical and Cognitive Differences Between SCD and NC

The 95 participants were divided into 46 SCD and 49 NC based on the AD8 score ≥ or <2 (Figure 1). There were no significant differences between groups in age, sex, and education level (Table 1). The SCD group had significantly higher sarcopenia risk and lower physical activity than the NC group in terms of the higher SARC-F score (1.15 ± 1.52 and 0.31 ± 0.62, *p* = 0.001), lower IPAQ-SF MET (3,288.52 ± 3,639.52 and 5,654.41 ± 4,397.93 MET min/week, *p* = 0.005), and less participants in the category of high IPAQ-SF (32.6 and 66.7%, *p* = 0.003). The SCD group performed worse than the NC group in both structured and individual cognitive tests with regard to the MoCA (23.87 ± 3.52 and 26.18 ± 3.52, *p* = 0.002), backward DST (6.00 ± 3.00 and 7.37 ± 2.81, *p* = 0.025), LNS (8.22 ± 2.46 and 9.61 ± 3.30, *p* = 0.024), animal CF (16.42 ± 4.81 and 19.02 ± 4.82, *p* = 0.010), color CF (12.22 ± 3.97 and 14.00 ± 4.56, *p* = 0.048), and FMT (33.94 ± 4.19 and 35.84 ± 4.84, *p* = 0.045) (Table 1).

**Table 1:** Comparisons between the SCD and NC groups.

	**SCD (*N* = 46)**	**NC (*N* = 49)**	***p***
**Basic information**			
Age	65.22 ± 5.44	66.04 ± 5.70	0.474
Sex (female)	30 (65.1 %)	25 (51.0 %)	0.161
Years of school education	9.28 ± 4.47	10.10 ± 3.95	0.345
AD8	3.78 ± 1.81	0.20 ± 0.41	<0.001^**^
**Physical activity**			
SARC-F	1.15 ± 1.52	0.31 ± 0.62	0.001^**^
SARC-F ≥ 4	4 (8.7 %)	0 (0.0 %)	0.051
IPAQ-SF MET (min/wk)	3288.52 ± 3639.52	5654.41 ± 4397.93	0.005^**^
IPAQ-SF category			0.003^**^
Low	11 (23.9 %)	6 (12.2 %)	
Middle	20 (43.5 %)	10 (20.4 %)	
High	15 (32.6 %)	33 (66.7 %)	
**Cognitive performance**			
*Structured cognitive test*			
MoCA	23.87 ± 3.52	26.18 ± 3.52	0.002^**^
MMSE	28.04 ± 1.65	28.33 ± 1.86	0.436
*Individual cognitive test*			
DSC	52.07 ± 17.17	59.88 ± 22.19	0.061
DST: forward	12.04 ± 2.37	12.39 ± 2.26	0.475
DST: backward	6.00 ± 3.00	7.37 ± 2.81	0.025^*^
LNS	8.22 ± 2.46	9.61 ± 3.30	0.024^*^
CF: animal	16.42 ± 4.81	19.02 ± 4.82	0.010^*^
CF: fruit	13.27 ± 3.30	14.04 ± 2.95	0.233
CF: color	12.22 ± 3.97	14.00 ± 4.56	0.048^*^
CF: city	19.16 ± 6.07	19.84 ± 5.64	0.574
FMT	33.94 ± 4.19	35.84 ± 4.84	0.045^*^

* *p < 0.05*.

** *p < 0.01*. *IPAQ-SF, international physical activity questionnaire-short form; MET min/wk, metabolic equivalent task minute per week; MoCA, Montreal cognitive assessment; MMSE, mini-mental state examination; DSC, digit-symbol coding; DST, digit span test; LNS, letter-number sequencing; CF, category fluency; FMT, facial memory test*.

### Spatial Differentiation of Physical- or Cognitive-Related White Matter in SCD

Thirty-one participants in the SCD group agreed to have DTI scans (Figure 1); they were all right-handed with mean age 65.13 ± 5.51 years, years of education 8.84 ± 4.17 years, and a female ratio of 74.2%. The DTI indices of different tracts had correlations to either physical activity or cognitive scores (Table 2). The physical activity IPAQ-SF MET had a positive correlation with the FA of right posterior corona radiata (PCR) by lower the physical activity metabolic equivalent, lower the white matter integrity (*r*_*s*_ = 0.501, *p* < 0.05 FDR corrected). Other tracts with marginal correlations with the IPAQ-SF MET were left PCR, bilateral posterior thalamic radiation, including optic radiation (PTR) and right pyramids segment of corticospinal tract (CST pyramids). The correlations between the SARC-F and the FA were marginal at right PCR and right external capsule (EC) (Table 2).

**Table 2:** Correlations of clinical and diffusion indices in SCD (*N* = 31), significance at *p* < 0.05.

**Measurement**	**Tract**	**Diffusion index**	**Correlation coefficient (*r_***s***_*)**	***p***
IPAQ-SF MET	CST pyramids (R)	FA	0.373	0.039
		MD	−0.389	0.031
	PCR (R)	FA	0.501	0.004^**^
	PCR (L)	FA	0.429	0.016
	PTR (R)	MD	−0.412	0.021
	PTR (L)	MD	−0.437	0.014
SARC-F	PCR (R)	FA	−0.432	0.015
	EC (R)	FA	−0.423	0.018
MoCA	ICP (R)	FA	0.463	0.009^*^
		MD	−0.642	<0.001^**^
	ICP (L)	FA	0.394	0.028
		MD	−0.527	0.002^**^
	CST pyramids (R)	MD	−0.533	0.002^**^
	Fornix (L)	MD	−0.468	0.008^*^
	AL-IC (L)	MD	−0.368	0.042
	PCR (R)	FA	0.375	0.038
	SCP (R)	MD	−0.414	0.021
	EC (R)	MD	−0.427	0.017
	Cin (L)	MD	−0.369	0.041
AD8	ICP (R)	MD	−0.374	0.038
	SFOF (L)	FA	0.363	0.045
	AL-IC (R)	MD	−0.415	0.020

*A correlation coefficient by Spearman's correlation coefficient (r_s_) in the 31 participants of the SCD group who agreed to undergo DTI study. This table summarized the statistically significant correlations at a confidence level of 95%*.

* *p < 0.01*.

** *A false discovery rate corrected p < 0.05*.

*r_s_, Spearman's correlation coefficient; SCD, subjective cognitive decline; IPAQ-SF MET, metabolic equivalent (minute per week) of the international physical activity questionnaire-short form; MoCA, Montreal cognitive assessment; R, right; L, left; CST pyramids, pyramids segment of the corticospinal tract; PCR, posterior corona radiata; PTR, posterior thalamic radiation, including optic radiation; EC, external capsule; ICP, inferior cerebellar peduncle; AL-IC, anterior limb of internal capsule; SCP, superior cerebellar peduncle; Cin, cingulum; SFOF, superior fronto-occipital fasciculus; FA, fractional anisotropy; MD, mean diffusivity*.

The cognitive-related white matter tracts were different from the physical activity-related tracts. The robust correlations between diffusion indices and the MoCA score were found at left inferior cerebellar peduncle (ICP) (MD *r*_*s*_ = − 0.527, *p* < 0.05 FDR corrected), right ICP (MD *r*_*s*_ = −0.642, *p* < 0.05 FDR corrected), and right CST pyramids (MD *r*_*s*_ = −0.533, *p* < 0.05 FDR corrected). The tracts of marginally significant microstructural correlations with the MoCA score distributed widely in bilateral hemispheres, including left fornix, left anterior limb of the internal capsule (AL-IC), right PCR, right superior cerebellar peduncle (SCP), right EC, and left cingulum (Cin) (Table 2).

The correlations between DTI index and the AD8 score were weak to moderate (Table 2) regarding the white matter of right ICP (MD *r*_*s*_ = −0.374, *p* = 0.038 uncorrected), left superior fronto-occipital fasciculus (SFOF) (FA *r*_*s*_ = 0.363, *p* = 0.045 uncorrected), and right AL-IC (MD *r*_*s*_ = −0.415, *p* = 0.020 uncorrected).

### Actigraphy-Related White Matter Integrity in SCD

Fifteen out of the 31 SCD participants further completed 72-h actigraphy recording. These 15 participants had a mean age of 66.33 ± 6.04 years, average years of education of 9.13 ± 4.26 years, and a female ratio of 73.4%. The Table 3 listed the tracts with significant white matter microstructure-actigraphy correlations at a confidence level of 99%. The intact white matter integrity of these tracts was associated with a good physical activity pattern by low intradaily variability and high interdaily stability. The FA of PCR had robust correlations with intradaily variability (right PCR *r*_*s*_ = −0.757, *p* < 0.05 FDR corrected; left PCR *r*_*s*_ = −0.875, *p* < 0.05 FDR corrected) and interdaily stability (right PCR *r*_*s*_ = 0.771, *p* < 0.05 FDR corrected). The FA of left superior corona radiata (SCR) significantly correlated with intradaily variability (*r*_*s*_ = −0.811, *p* < 0.05 FDR corrected).

**Table 3:** Correlations of diffusion indices and actigraphy in SCD (*N* = 15), significance at *p* < 0.01.

**Measurements**	**Tract**	**Diffusion**	**Correlation coefficient**	***p***
		**index**	**(*r_***s***_*)**	
Intradaily variability (IV)	PCR (R)	FA	−0.757	0.001^*^
	PCR (L)	FA	−0.875	<0.001^*^
	SCR (L)	FA	−0.811	<0.001^*^
Interdaily stability (IS)	PCR (R)	FA	0.771	0.001^*^

*The Spearman's correlation coefficients with p < 0.01 were considered statistically significant*.

* *A false discovery rate corrected p < 0.05. The actigraphy was recorded by the wearing device (SOMNOwatch^TM^) for 72 activities in 15 SCD participants*.

*r_s_, Spearman's correlation coefficient; SCD, subjective cognitive decline; FA, fractional anisotropy; PCR, posterior corona radiata; SCR, superior corona radiata*.

### Summary of the Correlations of Diffusion Indices and the Physical-Cognitive Decline in SCD

**Figure 3:**
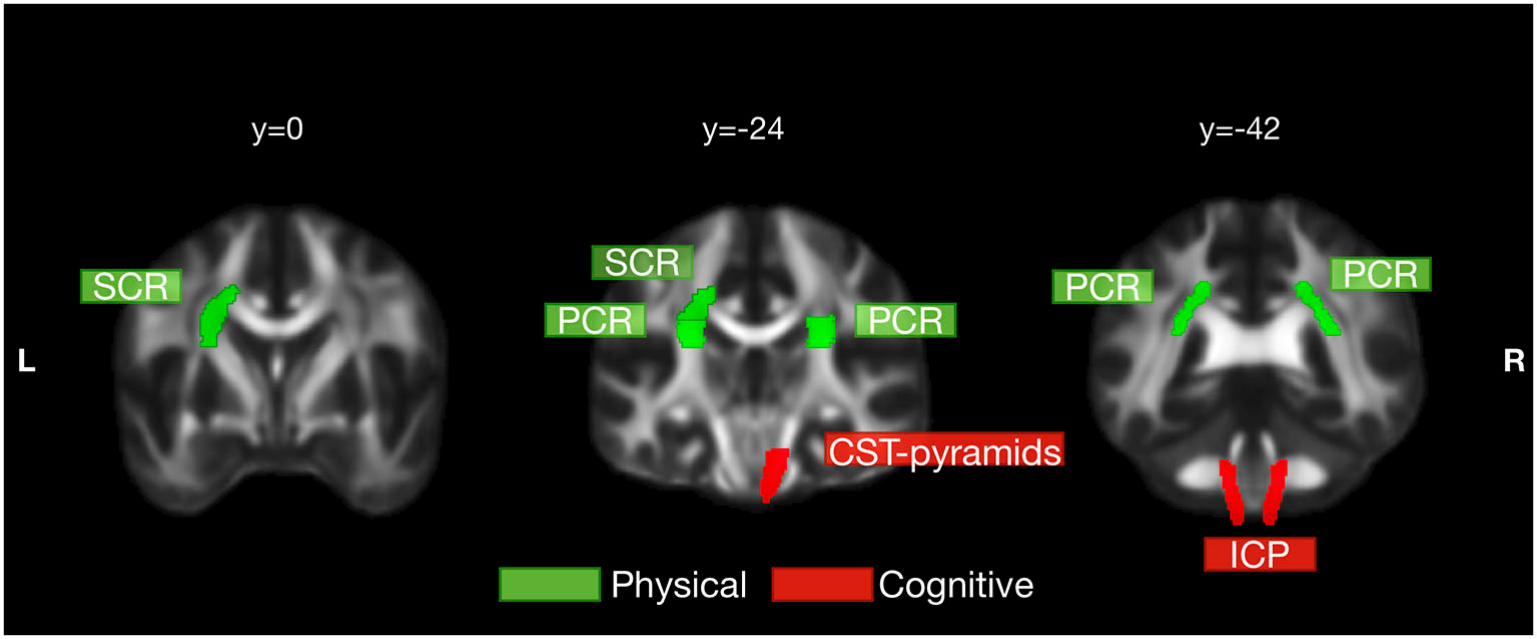
Physical activity- and cognition-related white matter tracts in SCD. White matter tracts that significantly correlated with physical activity and cognition were labeled in green and red, respectively. Physical activity was significantly correlated with left superior corona radiata (SCR) and bilateral posterior corona radiata (PCR). Cognitive performance was significantly correlated with bilateral inferior cerebellar peduncle (ICP) and right pyramids segment of corticospinal tract (CST pyramids). Right CST pyramids and right PCR had overlapped correlations with both physical activity and cognitive performance but still were more cognition- and physical activity-related, respectively. The tracts in this figure were those with at least one correlation coefficient at a FDR-corrected *p* < 0.05 to either physical activity or cognitive performance.

Table 4 and Figure 3 collected the tracts with at least one correlation coefficient at a FDR-corrected *p* < 0.05 to summarize the robust correlations between diffusion indices and clinical scores. Bilateral ICP, and right CST pyramids were cognition-related tracts with moderate correlations with the MoCA score (*r*_*s*_ ranged between 0.4 and 0.7). Right ICP notably correlated with both the MoCA and AD8 scores and might bridge between subjective and objective cognition. Several tracts were physical activity related, such as bilateral PCR and left SCR. Notably, right CST pyramids and right PCR had overlapped associations with physical activity and cognitive performance and highlighted potential mediation traits of white matter on the physical-cognitive co-decline in SCD.

**Table 4:** Summary of the tract-specific white matter integrity related to physical activity and cognition in SCD.

**Role**	**Tract**	**Cognitive**	**Physical**
		**correlations**	**activity correlations**
Cognition-related	ICP (R)^†^	MoCA (FA^*^/MD^**^)	
		AD8 (MD)	
	ICP (L)	MoCA (FA/MD^**^)	
	CST pyramids (R)	MoCA (MD^**^)	IPAQ-SF MET (FA/MD)
Physical activity- related	PCR (R)	MoCA (FA)	IPAQ-SF MET (FA^**^)
			SARC-F (FA)
			Actigraphy IV (FA^**^)
			Actigraphy IS (FA^**^)
	PCR (L)		IPAQ-SF MET (FA)
			Actigraphy IV (FA^**^)
	SCR (L)		Actigraphy IV (FA^**^)

*This table listed the tracts with at least one Spearman's correlation coefficient at a FDR-corrected p < 0.05*.

* *p < 0.01*.

** *A FDR-corrected p < 0.05*.

† *Associations with both subjective and objective cognition*.

*SCD, subjective cognitive decline; R, right; L, left; ICP, inferior cerebellar peduncle; CST pyramids, pyramids segment of corticospinal tract; PCR, posterior corona radiata; SCR, superior corona radiata; MoCA, Montreal cognitive assessment; IPAQ-SF MET, international physical activity questionnaire-short form, metabolic equivalent task minute per week; IV, intradaily variability; IS, interdaily stability; FA, fractional anisotropy; MD, mean diffusivity; FDR, false discovery rate*.

**Figure 4:**
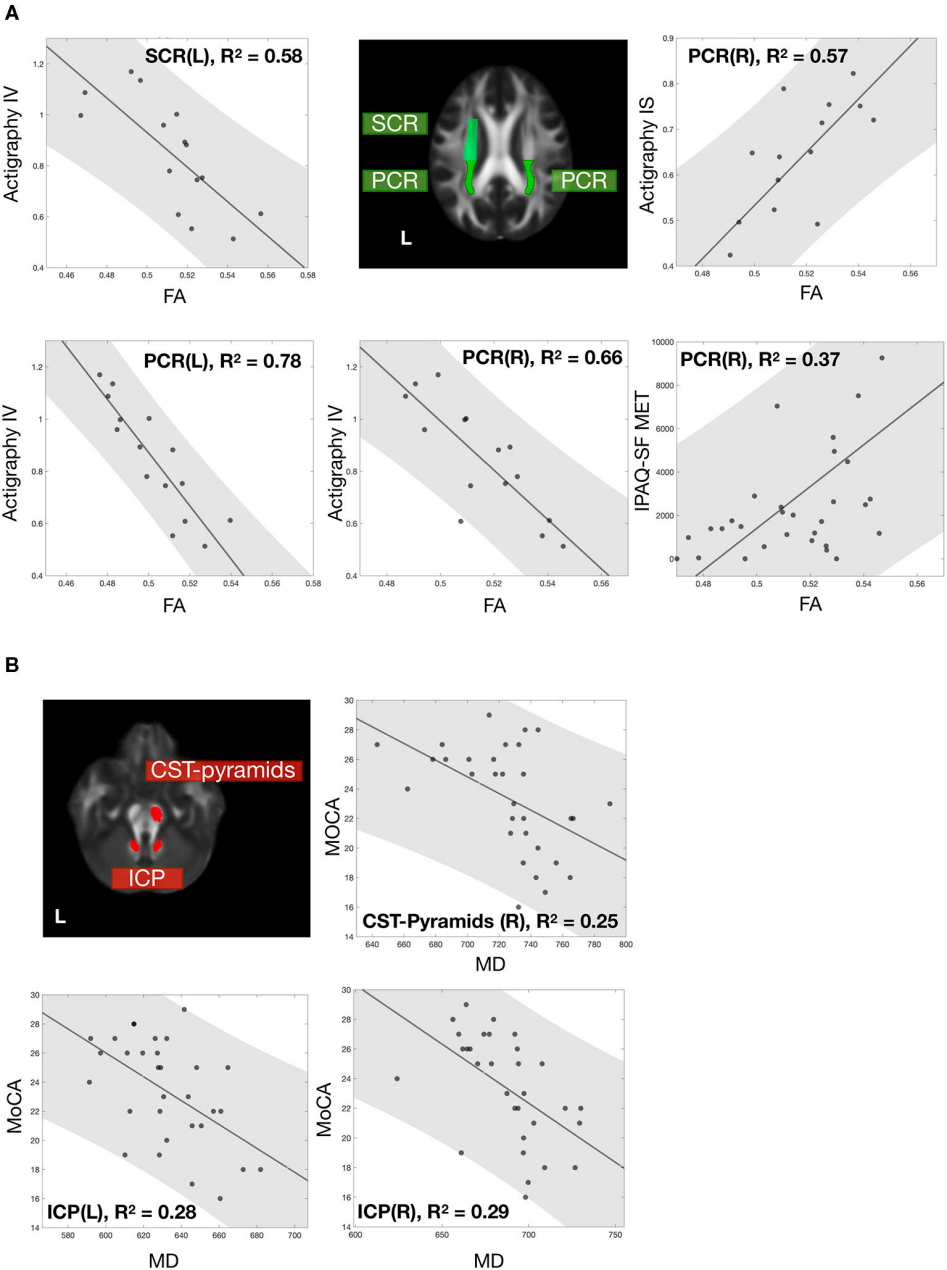
Correlations of white matter microstructure with physical activity and cognitive performance. The correlation between white matter microstructure and **(A)** physical activity and **(B)** cognitive performance. All data points were labeled in black dots, with a gray fitting line and a gray patch represent the 95% confidence interval. White matter tracts significantly correlated with physical activity and cognition and were labeled with green and red. All the correlations were significant with FDR-corrected *p* < 0.05. Noted that 15 participants who completed 72-h recording had actigraphy information; other assessments included all 31 participants.

In particular, FA values of bilateral PCR and left SCR were closely related to physical activity index. The intradaily variability of actigraphy was significantly correlated with the FA of left SCR and bilateral PCR. The actigraphy interdaily stability and the IPAQ-SF MET were also significantly associated with the FA of right PCR (Figure 4A). Unlike physical activity-related correlations, MD values of right CST pyramids and bilateral ICP correlated significantly with the MoCA score (Figure 4B).

To elucidate the diffusion-clinical correlations by eliminating the physical-cognitive interaction, partial correlation analysis controlled physical activity variables for the cognitive performance and vice versa. After correcting the cognitive-physical activity interaction, the partial correlation coefficients remained toward the same conclusion. The MD of bilateral ICP and right CST pyramids correlated with the MoCA score. The FA of bilateral PCR and left SCR was associated with the IPAQ-SF MET, SARC-F, actigraphy intradaily variability and interdaily stability. From this, the tracts were separated into cognition- and physical activity-related subgroups (Table 5).

**Table 5:** Partial correlation analysis of diffusion indices and clinical scales by controlling physical-cognitive interaction in SCD.

**Tract**	**Diffusion index**	***r_***p***_*: MoCA**			
**(a) Cognition-related partial correlation** ^**†**^					
ICP (R)	MD	−0.445^*^			
ICP (L)	MD	−0.443^*^			
CST-pyramids (R)	MD	−0.423^*^			
**Tract**	**Diffusion index**	***r**_***p***_* **: IPAQ-SF MET**	***r**_***p***_* **: SARC-F**	***r**_***p***_* **: Actigraphy IV**	***r**_***p***_* **: Actigraphy IS**
**(b) Physical activity-related partial correlation** ^**¶**^					
PCR (R)	FA	0.451^*^	−0.466^*^	−0.752^**^	0.610^*^
PCR (L)	FA	0.430^*^		−0.867^***^	
SCR (L)	FA			−0.811^**^	

*The partial correlation analysis tested the tracts in Table 4. This table only embraced the statistically significant partial correlations (p < 0.05, uncorrected). During partial correlation analysis, the cognitive-related diffusion indices were corrected with the diffusion indices of the physical activity-related tracts, and vice versa*.

* *p < 0.05*.

** *p < 0.01*.

*** *p < 0.001*.

† *Controlled variables were the MD values of PCR (R), PCR (L), and SCR (R)*.

¶ *Controlled variables were the FA values of ICP (R), ICP (L), and CST pyramids (R)*.

*r_p_, partial correlation coefficient; IV, intradaily variability; IS, interdaily stability; PCR, posterior corona radiata; SCR, superior corona radiata; ICP, inferior cerebellar peduncle; CST pyramids, pyramids segment of the corticospinal tract; L, left; R, right; FA, fractional anisotropy; MD, mean diffusivity*.

## Discussion

### Physical-Cognitive Co-decline in Older Adults

During normal aging, maintaining high cognitive function requires three main factors: intellectually engaging activities, social engagement, and physical activities (Harada et al., 2013). Persistently physically active states in young and older age help cognitive reserve according to the cognitive trajectory observation in a community-dwelling population (Reas et al., 2019). In the Framingham Study, people with high physical activity index were free from Alzheimer's disease and brain atrophy (Tan et al., 2017). In abnormal aging, frailty is composed of two primary parts: physical and cognitive (Kelaiditi et al., 2013; Brigola et al., 2015). According to a meta-analysis, physical frailty is associated with cognitive status in the elderly (Furtado et al., 2018). The presence of physical frailty predicts subsequent cognitive decline (Borges et al., 2019). Cognitive impairment has been proposed to be a component of frailty syndrome (Houles et al., 2012), in addition to the five typical Fried criteria phenotypes of physical frailty: weakness, exhaustion, slowness, low activity, and unintentional weight loss (Fried et al., 2001). The coexistence of physical weakness and cognitive impairment is named “cognitive frailty” (Kelaiditi et al., 2013) or “physio-cognitive decline syndrome” (Liu et al., 2020), representing cognitive vulnerability of physically weakened people. The physical-cognitive reduction is also considered a phenotype of accelerated aging (Chen and Arai, 2020) or a reduced physiological reserve, pushing the aging people away from homeostasis (Clegg et al., 2013).

Subjectively detected cognitive impairment is gaining importance as an early sign of inadequate cognitive reserve to deal with daily cognitive requirements. SCCs also correlate with physical frailty and interact in between the physical-cognitive decline (Gifford et al., 2019). In line with the above knowledge, this study further revealed physical inactivity as a mediator between the subjective and objective decline of cognition. Self-awareness of cognitive well-being requires good physical activity to maintain satisfactory cognitive functioning. The older adults with SCD have less active physical status and lower cognitive performance than the age- and education-equivalent NC.

### White Matter Changes Underlie the Physical-Cognitive Decline in SCD

Previous studies suggested SCD being an early form of dementia according to longitudinal observations (Hohman et al., 2011; Mitchell et al., 2014; Kaup et al., 2015; Koppara et al., 2015), neuropathological evidence (Amariglio et al., 2015b; Buckley et al., 2017), and genomic studies (Moreno-Grau and Ruiz, 2016). Destruction of white matter microstructure develops as early as cognitive changes only at the subjective level and before neuronal loss could be measured (Selnes et al., 2012). The reduced white matter integrity is also associated with worse performance in general cognition, memory (Luo et al., 2019), and executive function (Ohlhauser et al., 2019) in SCD.

Physical conditions have their unique corresponding white matter in the brain. Previous studies showed evidence that physical deterioration and white matter degeneration develop side by side. Being physically active predicts the preservation of white matter integrity at the genu of corpus callosum, uncinate, EC, and AL-IC (Strommer et al., 2020). White matter volume reduces alongside the decrease of physical fitness and activity (Sexton et al., 2016). Loss of white matter integrity predicts development and progression of physical frailty (Maltais et al., 2020). Increased hyperintensities of white matter are also concomitant with the decline of physical frailty (Avila-Funes et al., 2017).

In this study, diffusion imaging in the SCD group further revealed the association of white matter microstructure and physical activity, which were partially overlapped and partly distinguishable from the cognitive-related white matter tracts (Table 4 and Figure 3). The CST pyramids white matter microstructure was related to both physical activity and cognitive performance. It, therefore, may be the critical tract responding for the mediation effects of physical activity between subjective and objective cognition. The PCR white matter integrity was mainly correlated with physical activity and marginally associated with the cognitive score. On the contrary, integrity of ICP white matter affected both SCCs and objective cognitive performance but had minor roles with physical activity. It may be the underlying microstructure basis of subtle cognitive decline in SCD. To summarize, the degenerative changes of white matter develop in tract-specific ways for physical and cognitive representations in preclinical dementia.

Notably, the diffusion index FA values were strongly correlated with multiple indicators of physical activity, while MD changes were tightly associated with the MoCA score (Tables 4, 5). FA represents the degree of anisotropy of water molecule diffusion. The degenerative processes, like demyelination or apoptosis, may lower FA by removing barriers to the perpendicular diffusion and, usually, be deemed the cause of the decline in white matter integrity (Beaulieu, 2002; De Lange et al., 2016). On the other hand, MD is the average of three eigenvalues of water diffusivity. Damage to the tissue may cause the increase of free diffusion, resulting in higher MD (Basser, 1995; Soares et al., 2013). Previous studies indicated that the degeneration of white matter usually induces a decrease of FA and an increase of MD (Alexander et al., 2007). More importantly, our result showed a clear trend that reduced and worsened patterns of physical activity (higher variability and lower stability) were negatively correlated with FA, which may suggest lower fiber integrity in those physical-related white matter tracts. Furthermore, the cognitive performance was negatively correlated with MD, which may infer damage in cognitive-related white matter tracts.

### Clinical Significance of the Corticospinal and Corona Radiata White Matter

The diffusion indices of right CST pyramids and right PCR had correlations with both the MoCA score and physical activity indices. The white matter integrity of right CST pyramids and right PCR might maintain the physical activity-mediated cognitive preservation in SCD. The correlations were more robust to cognitive performance in right CST pyramids and more robust to physical activity in right PCR. These differences might reflect their weights on mediation effects between physical activity and cognitive performance. We also noticed that these two tracts were both on the right hemisphere. A lateralization shift could occurred in neurodegeneration. In MCI and Alzheimer's disease, rightward lateralization of functional connectivity suggested increasing compensatory efforts at the non-dominant hemisphere to compensate for the functional loss of the dominant left hemisphere (Liu et al., 2018). However, white matter degeneration failed to show lateralized differences of FA and MD in DTI studies (Sexton et al., 2011). In this study, we only enrolled right-handed participants whose dominant hemisphere was on the left side. The roles of right PCR and CTS pyramids in physical-cognitive mediation might be compensatory functional maintenance of the non-dominant hemisphere (Table 4). However, the PCR and ICP at both sides involved physical and cognitive correlations after removing physical-cognitive interactions (Table 5). The lateralized discrepancy of white matter's clinical correlations was not significant in this study.

PCR was the single tract with significantly lower FA in amnestic MCI than healthy controls, as shown by a meta-analysis (Yu et al., 2017). In longitudinal tracking of white matter heterogenicity in an aging population, the PCR was one of the specific tracts that identified individuals at risk of cognitive decline (Poulakis et al., 2021). In this study, the importance of PCR's white matter integrity in maintaining cognition shifted to an earlier preclinical stage. Therefore, we propose that PCR is a key region of white matter destruction in the early cognitive decline of the Alzheimer's disease spectrum; its cognitive influence is mediated by physicalactivity.

In the Rotterdam Study, which enrolled a large number of non-demented participants, the MD of CST was negatively associated with global cognition and individual tasks (Letter-Digit Substitution Test and Stroop), involving attention, visuomotor coordination, psychomotor speed, cognitive flexibility, and executive functions (Cremers et al., 2016). The CST determines psychomotor speed by controlling the non-decision time for the latencies of stimulus encoding and action initiation (Karahan et al., 2019). White matter microstructural destruction of CST developed in multiple-domain amnestic MCI (Li et al., 2013) and moderate-to-severe Alzheimer's disease (Canu et al., 2010). Although once an unexpected finding (Li et al., 2013), the CST is no longer merely motor fiber bundles but also considered a cognition-related tract. Our results further suggested that CST influences cognitive performance partly through physical activity in preclinical dementia.

Notably, the CST in the JHU ICBM DTI-81 atlas contains only the brainstem segment (pyramids) of CST, while the PCR comprises part of the corona radiata segment of CST. Therefore, CST along the supratentorial and the infratentorial routes possesses the mediating traits of physical-cognitive reserve in SCD. In a study of young, healthy people, the FA of widespread white matter positively correlated with physical fitness and mediated between walking endurance and cognitive performance; although the probabilistic mapping of the decisive white matter mostly fell in unclassified areas of the JHU ICBM DTI-81 atlas, this study recognized the CST-containing white matter skeletons, including the CST pyramids, SCR, PCR, and cerebral peduncles, as the physical-cognitive mediating tracts (Opel et al., 2019). Similarly, our study of older adults with SCD also located the physical-cognitive-related white matter at CST-containing regions, the right PCR and right CST pyramids of the JHU ICBM DTI-81 atlas.

### Cognitive Roles of the Olivo-Cerebellar White Matter in SCD

In recent 30 years, viral tracers of neural tract-tracing and progress of functional neuroimaging overcame the limitations of conventional tracing techniques in studying the polysynaptic nerve fibers and revealed the non-motor functions of the cerebellum (Buckner, 2013). In 1991, Schmahmann hypothesized the roles of cerebellum in cognitive control that, “in the same way as the cerebellum regulates the rate, force, rhythm, and accuracy of movements, so may it regulate the speed, capacity, consistency, and appropriateness of mental or cognitive processes” (Schmahmann, 1991). The parallels of similar microzones, the *modules*, of parasagittal stripes of Purkinje cells receive climbing fibers from inferior olive, project fibers to deep cerebellar nuclei, and then feedback to the inferior olive to make a loop to support consistent computational operations of the cerebellum (Ruigrok, 2011; Apps et al., 2018). The cerebellar modules further project to contralateral cerebral sensorimotor and association cortices in a neat topographic arrangement (Buckner et al., 2011; Palesi et al., 2017; King et al., 2019) and anticipatorily process multiple higher cortical functions (D'Angelo and Casali, 2012). The cerebro-cerebellar feed-forward loops made the cerebelluma a *forward controller* for motor and non-motor predictions (D'Angelo and Casali, 2012; Schmahmann et al., 2019).

The neural basis of cerebellar modules for *universal cerebellar transform* (Schmahmann et al., 2019) is the olivo-cortico-nuclear circuitry between the inferior olive, Purkinje cells, and deep cerebellar nuclei (D'Angelo and Casali, 2012; Apps et al., 2018). The ICP is the main entrance of the olivocerebellar tract to the cerebellum and maintaining the functioning of the cerebro-cerebellar circuits (Luo and Sugihara, 2016). Atrophy of ICP could be severe in spinocerebellar ataxia 6 (Wang et al., 2010), a relatively pure cerebellar-involved type of inherited spinocerebellar ataxic disorder, and present as prefrontal dysfunction (Suenaga et al., 2008; Kawai et al., 2009). In this study, white matter integrity of the bilateral ICPs was crucial in cognitive preservation in SCD and was also associated with subjective cognition. Diffusion indices of the ICP were purely cognitive and not related to physical activity. Our results suggested that white matter integrity of ICP directly affected the cognitive performance of SCD and did not underlie the physical inactivity-mediated cognitive decline. In previous MRI studies of Alzheimer's disease spectrum, the gray matter atrophy, white matter dysintegrity, or functional changes of the cerebellum were usually considered incidental findings. However, based on the *dysmetria of thought* theory (Schmahmann et al., 2019), the importance of the cerebellum in cognitive decline was supported by revisiting previous findings (Jacobs et al., 2018) and our results. The white matter health of the cerebellum could be a biomarker, indicating one's cognitive trajectory.

### Limitations

This study has several limitations. The first to be considered is the selection bias. Because whether to join the study depended on willingness of individuals, those with active personality types, good physical status, and cognitive well-being might be unintentionally recruited. Second, no longitudinal follow-up data to show trajectories of cognitive decline. Therefore, follow-up of the participants is warranted to trace the interconnected relationship among subjective and objective cognition, physical activity, and the underlying white matter integrity. Third, this study population was limited to SCD. No comparative group of MCI or dementia limits its generalization to Alzheimer's disease spectrum as other studies did (Wang et al., 2012; Doan et al., 2017). Contrary to other SCD studies that primarily enrolled the patients visiting the memory clinics for their memory decline worries, this study recruited participants from communities and found them mainly normal or at the SCD stage. Although limited in generalizing to advanced dementia stages, our results were advantageous in generalizing to the general population. Fourth, the MRI analysis in this study did not include a control group and could not conclude if the white matter changes were specific for SCD. We could not exclude the possibilities of aging effects from neurodegeneration-related white matter changes in the current study setting. A follow-up study is warranted to compare the white matter changes in the SCD with the control group. Fifth, not all the participants receiving MRI scans or recording actigraphy reduced the power of correlation analysis. An independent validation cohort or recruiting more participants could increase the power of the results and minimize the potential overfitting problem in future work. Finally, the diffusion protocol in this study only has a single b-shell, preventing the use of diffusion models that are more sophisticated than DTI. Although DTI has been widely used to study brain white matter, its capacity of evaluating white matter microstructure was limited and must be interpreted with caution (Jones et al., 2013). It would be necessary to utilize more advanced models with a multi-shell protocol to have a finer analysis of white matter changes in SCD in the future studies.

## Conclusions

Physical inactivity mediated between self-awareness of cognitive decline and measurable decrease of cognitive performance in community-dwelling cognitively normal older adults. Coupling of the physical-cognitive co-decline occurred in the SCD when compared with NC. Physical activity and cognitive performance had their corresponding spatial-specific white matter in SCD. The white matter integrity of right PCR and right CST at the pyramids segment was cross-related to physical and cognitive indices and might explain the physical activity-mediated cognitive decline in SCD. Contrarily, cerebellar white matter integrity at bilateral ICP correlated explicitly with objective cognition. As the results suggested, cognitive maintenance in preclinical dementia might rely on olivo-cerebellar system through intactness of ICP microstructure and subsequently intact cerebro-cerebellar modulation.

## Data Availability Statement

The raw data supporting the conclusions of this article will be made available by the authors, without undue reservation.

## Ethics Statement

The studies involving human participants were reviewed and approved by Institutional Review Board of Chang Gung Memorial Hospital. The patients/participants provided their written informed consent to participate in this study.

## Author Contributions

Y-CW: conceptualization, methodology, data curation, formal analysis, investigation, resource, writing—original drafting, visualization, project administration, and funding acquisition. C-CHH: methodology, software, formal analysis, writing—original drafting, and visualization. W-YH: resource, data curation, and funding acquisition. Y-LC: data curation and supervision. CheminL: methodology and resource. C-KC: resource, supervision, and project administration. ChenL: formal analysis. Y-CS: data curation and resource. C-PL: conceptualization, methodology, investigation, resource, writing, review, editing, and supervision. All authors contributed to the article and approved the submitted version.

## Conflict of Interest

The authors declare that the research was conducted in the absence of any commercial or financial relationships that could be construed as a potential conflict of interest.

## Publisher's Note

All claims expressed in this article are solely those of the authors and do not necessarily represent those of their affiliated organizations, or those of the publisher, the editors and the reviewers. Any product that may be evaluated in this article, or claim that may be made by its manufacturer, is not guaranteed or endorsed by the publisher.
